# Radiographic Risk Factors for Contralateral Rupture in Dogs with Unilateral Cranial Cruciate Ligament Rupture

**DOI:** 10.1371/journal.pone.0106389

**Published:** 2014-09-25

**Authors:** Connie Chuang, Megan A. Ramaker, Sirjaut Kaur, Rebecca A. Csomos, Kevin T. Kroner, Jason A. Bleedorn, Susan L. Schaefer, Peter Muir

**Affiliations:** Comparative Orthopaedic Research Laboratory, and the Department of Surgical Sciences, School of Veterinary Medicine, University of Wisconsin-Madison, Madison, Wisconsin, United States of America; The Ohio State University, United States of America

## Abstract

**Background:**

Complete cranial cruciate ligament rupture (CR) is a common cause of pelvic limb lameness in dogs. Dogs with unilateral CR often develop contralateral CR over time. Although radiographic signs of contralateral stifle joint osteoarthritis (OA) influence risk of subsequent contralateral CR, this risk has not been studied in detail.

**Methodology/Principal Findings:**

We conducted a retrospective longitudinal cohort study of client-owned dogs with unilateral CR to determine how severity of radiographic stifle synovial effusion and osteophytosis influence risk of contralateral CR over time. Detailed survival analysis was performed for a cohort of 85 dogs after case filtering of an initial sample population of 513 dogs. This population was stratified based on radiographic severity of synovial effusion (graded on a scale of 0, 1, and 2) and severity of osteophytosis (graded on a scale of 0, 1, 2, and 3) of both index and contralateral stifle joints using a reproducible scoring method. Severity of osteophytosis in the index and contralateral stifles was significantly correlated. Rupture of the contralateral cranial cruciate ligament was significantly influenced by radiographic OA in both the index and contralateral stifles at diagnosis. Odds ratio for development of contralateral CR in dogs with severe contralateral radiographic stifle effusion was 13.4 at one year after diagnosis and 11.4 at two years. Odds ratio for development of contralateral CR in dogs with severe contralateral osteophytosis was 9.9 at one year after diagnosis. These odds ratios were associated with decreased time to contralateral CR. Breed, age, body weight, gender, and tibial plateau angle did not significantly influence time to contralateral CR.

**Conclusion:**

Subsequent contralateral CR is significantly influenced by severity of radiographic stifle effusion and osteophytosis in the contralateral stifle, suggesting that synovitis and arthritic joint degeneration are significant factors in the disease mechanism underlying the arthropathy.

## Introduction

Complete cranial cruciate ligament rupture (CR) is an important cause of stifle instability and associated pelvic limb lameness in dogs in which fiber damage to the caudal cruciate ligament is also common [1], [2]. Each year, at least one billion dollars are spent in the United States on treatment of CR and associated meniscal tearing [3]. While CR can result from trauma, a large majority of dogs develop CR during normal activity in association with pre-existing degeneration of the stifle joint and the cruciate ligament complex [4], [5]. Among dogs presented with unilateral CR, a large proportion of patients will develop contralateral CR within 12 to 24 months of initial diagnosis [6], [7]. In previous work, analysis of this risk has been reported as an incidence after surgery (percentage of patients within the cohort). This risk is in the range of 22–54% at 6 to 17 months of diagnosis [6], [8]–[11]. More recently survival analysis has been used to evaluate risk factors for development of contralateral CR [7], [12].

Many studies have investigated the disease mechanism and examined clinically relevant markers of disease or risk factors for development of CR. A current hypothesis relevant to the CR disease mechanism is that stifle joint inflammation precedes development of stifle instability from CR. Development of synovitis is an early event in the incipient phase of the condition that precedes development of clinically detectable joint instability, based on arthoscopic examination of the stifle [2]. Development of stifle synovitis also increases the risk of subsequent contralateral CR in dogs [13].

Moderate to severe osteoarthritis (OA) is usually detectable radiographically in the affected index stifle at the time of diagnosis of unilateral CR [14], [15]. Radiographic signs of OA are often present in clinically stable contralateral stifle joints at the time of diagnosis [2], [12], [14], [15], but underestimate severity of synovitis [2]. It has been recognized for some time that radiographic signs of contralateral stifle joint degeneration in dogs with unilateral CR influence risk of contralateral CR [6], [9]. However, these analyses were limited to determining that global scoring of radiographic change, including synovial effusion and osteophytosis, influenced risk of contralateral CR.

In a previous experiment, survival analysis of time to contralateral CR in a large group of dogs presented with unilateral CR was conducted using cases from three referral centers to examine the pattern of contralateral CR in the affected population studying a variety of surgical stabilizing treatments [7]. The median time to contralateral CR for the entire affected population was 947 days [7]. However, survival analysis in this previous study did not consider radiographic change or type of surgical treatment at the time of diagnosis in the statistical model. Past research suggests that both synovial effusion and osteophytosis assessed radiographically would significantly influence time to contralateral CR. More recently, the presence of radiographic synovial effusion and osteophytosis in the contralateral stifle at diagnosis of unilateral CR has been shown to be a significant risk factor for development of contralateral cruciate rupture over time [12], supporting the concept that radiographic abnormalities at the time of initial diagnosis are predictive of clinical outcome. Functional outcome after treatment of CR with a stabilizing surgical procedure is procedure-dependent. Consequently, surgical procedure could also influence time to contralateral CR. Functional outcome at 12 months after treatment with tibial plateau leveling osteotomy (TPLO) is superior to treatment with a lateral fabellar suture [16], [17], and meta-analysis of surgical treatments suggests that evidence most strongly supports the ability of TPLO to return dogs to normal function [18].

The present study aimed to evaluate severity of radiographic change in the unstable index and stable contralateral stifle joints at the time of diagnosis of unilateral CR and examine synovial effusion and osteophytosis as risk factors for contralateral CR. We hypothesized that risk of contralateral CR would be significantly influenced by the severity of synovial effusion and osteophytosis in both stifles at the time of initial diagnosis of unilateral CR. Confirmation that radiography is an important predictive marker for risk of subsequent contralateral CR could be relevant to clinical management of affected dogs. We designed this study to provide baseline date for ongoing longitudinal studies of disease-modifying treatment for the underlying arthropathy.

## Materials and Methods

### Dogs

**Figure 1:**
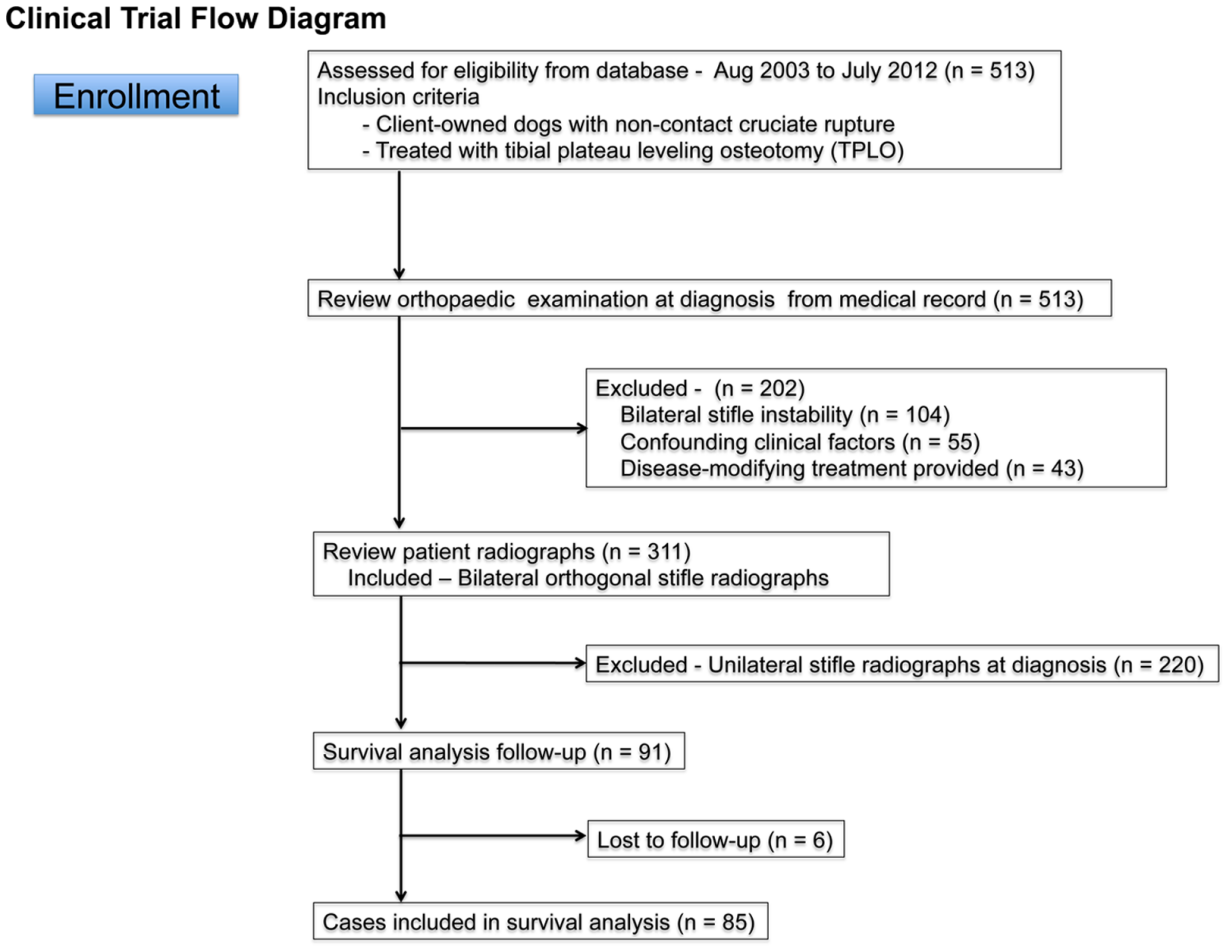
Flow diagram for case inclusion and exclusion. Of the 513 cases identified from the initial medical records search, 85 dogs were ultimately included in the survival analysis.

Medical records of dogs that were admitted to the Small Animal Orthopaedic Surgery Service at the University of Wisconsin-Madison UW Veterinary Care Hospital for unilateral (TPLO) treatment of CR between Aug 2003 and July 2012 were reviewed. Because functional outcome after treatment of CR with a stabilizing surgical procedure is procedure-dependent and meta-analysis of surgical treatments suggests that evidence most strongly supports the ability of TPLO to return dogs to normal function [16]–[18], the study was limited to TPLO-treated dogs to minimize any variation in time to contralateral CR associated with different surgical treatments. The initial medical record search identified 513 dogs that were treated with TPLO. To be included in the study, dogs had to meet the following criteria: Diagnosis of unilateral CR with radiographs of both stifles made at the time of diagnosis. Dogs were excluded for the following reasons: Diagnosis of bilateral CR, unilateral stifle radiographs at the time of diagnosis, the presence of confounding clinical factors that could affect patient mobility and a normal convalescence after surgery, and lack of clinical follow-up. Medical records from 428 dogs were excluded after filtering (**Fig. 1**). Records from 85 dogs were then reviewed in detail as described below.

### Medical Records Review and Diagnosis of Cranial Cruciate Ligament Rupture

Diagnosis of CR was defined by the detection of stifle instability clinically. CR was confirmed by physical examination and detection of cranial tibial translation using the cranial drawer and cranial tibial thrust tests [19], as well as assessment of periarticular fibrosis and joint thickening. Age, gender, breed, and body weight for the study cohort were obtained from the medical record.

### Radiography

**Figure 2:**
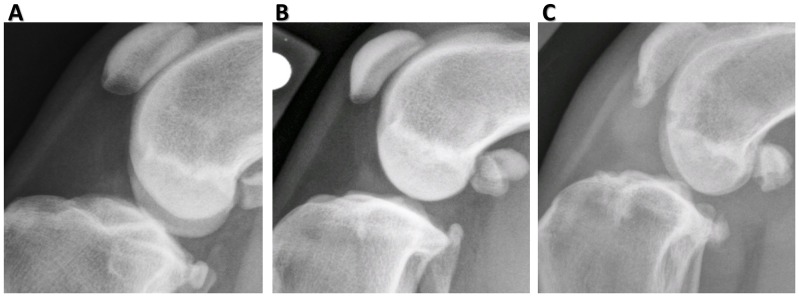
Severity scoring of stifle radiographs for synovial effusion. (**A–C**) Severity of synovial effusion was graded as 0 = normal (A), 1 = mild (B), or 2 = severe (C), using the medial-lateral radiographic view. Severity scoring was based on the magnitude of the soft tissue density within the stifle joint and the dimensions of the intra-patellar fat pad density in the cranial part of the joint. Grading was based on a previously published scale [15].

**Figure 3:**
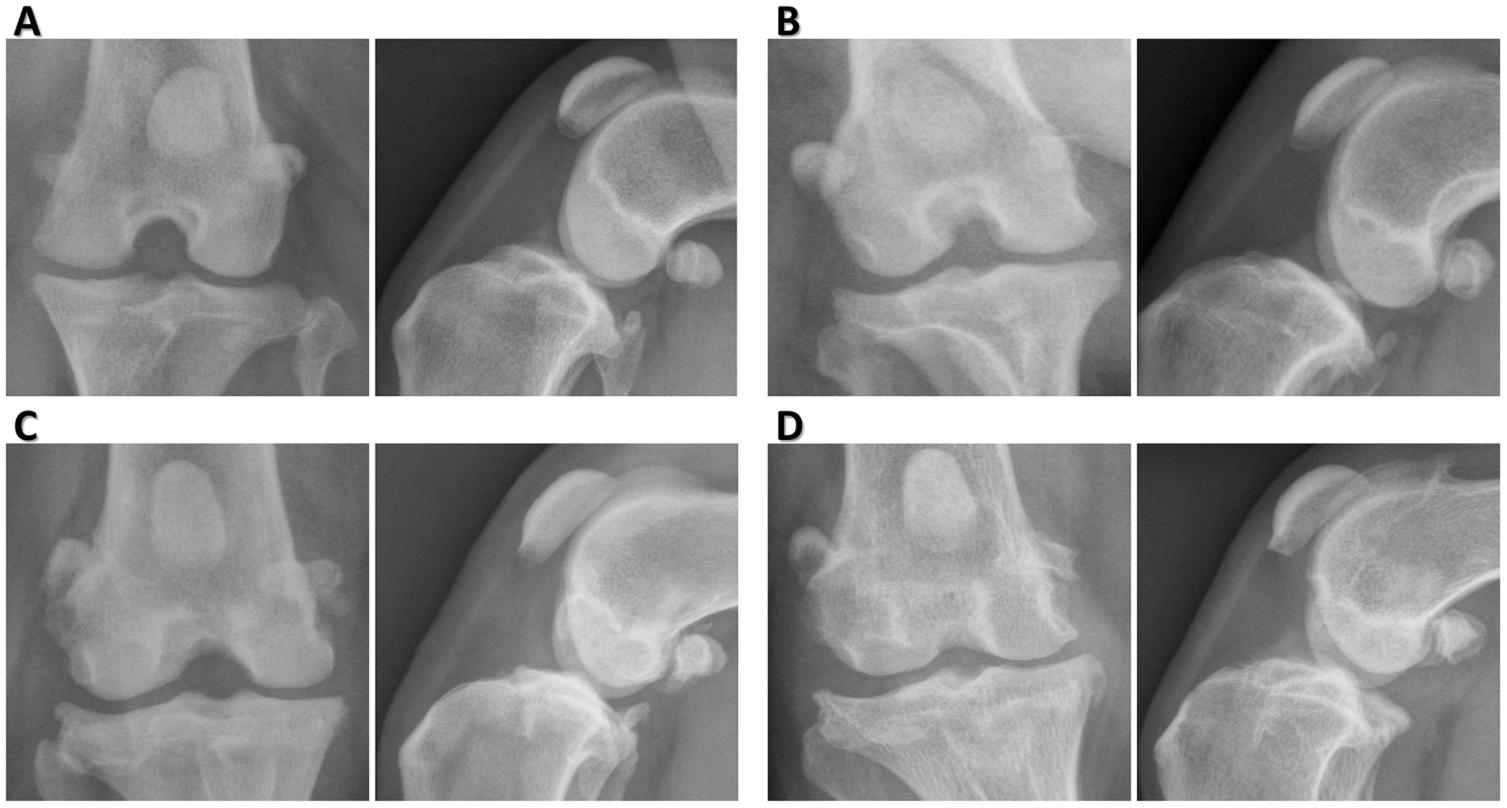
Severity scoring of stifle radiographs for osteophytosis. (**A–D**) Severity of osteophytosis was graded as 0 = normal (A), 1 = mild (B), 2 = moderate (C) or 3 = severe (D) respectively after evaluation of orthogonal views of the stifle. Severity scoring was based on the magnitude and severity of osteophyte formation around the joint margins, including the proximal and distal poles of the patella, the lateral and medial aspects of the trochlear ridges of the distal femur, and the lateral, medial, cranial, and caudal aspects of the proximal tibia, and the fabellae. Grading was based on a previously published scale [15].

Synovial effusion and osteophytosis were used to evaluate the severity of stifle OA. These criteria were selected based on high reproducibility [15], [20]. The most important early radiographic sign of stifle OA is development of synovial effusion and associated compression of the infrapatellar fat pad [14]. Orthogonal cranio-caudal and medio-lateral radiographic views of the index stifle that was treated with TPLO were reviewed. Radiographic views of the contralateral stifle were similarly reviewed. Synovial effusion was graded subjectively on a scale from 0–2 (0 - normal, 1 - mild, 2 - severe) (**Fig. 2**). Both cranial and caudal stifle joint spaces were examined in this assessment. Cranially, the extent of effusion and the shape of the infrapatellar fat pad were considered. Caudal bulging of the joint capsule was also evaluated during grading. Osteophytosis was graded subjectively on a scale from 0–3 (0 - normal, 1 - mild, 2 - moderate, 3 - severe) based on the severity of osteophytosis at the margins of the stifle joint (**Fig. 3**). Grading was based on previously described numerical rating scales [15]. Radiographic scoring was performed by a single observer (CC), after training was provided by an experienced clinician (PM). In addition, the tibial plateau angle (TPA) was calculated from the lateral radiographic views of the index and contralateral tibias with the stifle and tarsus held in ninety degrees of flexion. For lateral radiographs which did not include the entire tibia, a longitudinal reference axis as long as possible was used [21].

### Clinical Follow-Up

Clinical follow-up was done by medical record review, telephone conversation with the owner of the dog or the primary veterinarian, or orthopaedic examination at the UW Veterinary Care Hospital. The presence of contralateral CR was confirmed by detection of stifle instability by a veterinarian. During telephone follow-up with owners, the presence or absence of contralateral pelvic limb lameness and any relevant clinical findings made by their primary veterinarian were determined. Dogs that had experienced contralateral CR were coded as a complete case and time from initial diagnosis to contralateral CR was calculated in days. If an individual dog had not experienced contralateral CR at time of follow-up, the case was coded as censored and the time from initial diagnosis to clinical follow-up was calculated in days. If the dog did not return to the UW Veterinary Hospital after surgery or the owner was lost to follow-up, then the case was excluded from the analysis.

### Statistical Analysis

Data were reported as mean ± standard deviation or median (range) as appropriate. The Student’s *t* test for paired data was used to compare TPA in index and contralateral stifles. The Spearman Rank test was used to determine whether severity of synovial effusion and osteophytosis in index and contralateral stifles were correlated and whether clinical parameters were correlated with radiographic change. Precision of radiographic scoring for synovial effusion and arthritis degeneration of the stifle was determined. One observer (RAC) evaluated a series of orthogonal stifle radiographs from 15 dogs that represented the range of severity for synovial effusion and osteophytosis three times in a blinded fashion to determine intra-observer repeatability of the scoring system using the intraclass correlation coefficient (ICC) statistic. The series of radiographs from 15 dogs were also graded by two other observers (CC, KTK) in a blinded fashion. Collectively, these observations were used to determine inter-observer reproducibility of the scoring system using the ICC. ICC ≤0.3 were considered weak, coefficients >0.3 and <0.75 were considered moderate, and ≥0.75 were considered strong.

Data from clinical follow-up of the cohort was used for survival analysis. Contingency tables for radiographic grading of severity of synovial effusion and osteophytosis in both the index and contralateral stifle were made for development of contralateral CR at one year and at two years after diagnosis and treatment of the index stifle with TPLO. The effect of severity of synovial effusion and osteophytosis of the index and contralateral stifle joints on development of contralateral cruciate rupture was examined using a Monte-Carlo simulation Chi-square test [22]. For tables with a significant disease association, Chi-square or Fisher exact testing, as appropriate, was used to determine which grades of synovial effusion or osteophytosis had significant effects on time to contralateral CR. Odds ratios and 95% confidence limits were also calculated for each grade.

Logistic regression and Cox’s Proportional Hazards models were also used to determine which clinical factors might influence risk of contralateral CR. Initially, putative risk factors were analyzed in a univariate model. Factors considered in the univariate model included age, gender, body weight, dog breed, TPA, and severity of synovial effusion and osteophytosis in the index and contralateral stifles. Univariate parameters with *p*<0.2 were then considered further in a multivariate model. Survival curves stratified by radiographic scoring of synovial effusion and osteophytosis severity were also compared using the Logrank (Mantel-Cox) test. All results were considered significant at *p*≤0.05.

## Results

### Study Cohort

The initial medical records search identified 513 dogs treated with TPLO. Case filtering excluded 428 dogs. Dogs with bilateral stifle instability at diagnosis were excluded (n = 104). Dogs with confounding factors that could affect patient mobility, normal weight bearing, or a normal outcome from TPLO surgery were excluded (n = 55, **Table 1**). Furthermore, dogs given medical treatments after surgery that could be disease-modifying were also excluded (n = 43). These treatments included bilateral arthroscopy with associated joint lavage, leflunomide treatment and doxycycline treatment. Radiographs for the remaining 311 dogs were then reviewed. Of these 311 dogs, 220 were excluded because bilateral orthogonal radiographic views at diagnosis were lacking, and 6 dogs were lost to follow-up. After the filtering process, data from 85 dogs were available for survival analysis (**Fig. 1**).

**Table 1:** Confounding factors for case exclusion.

Confounding factor	Number of Dogs
Previous stifle surgery	30
TPLO treatment for a stable partial cruciate rupture	9
TPLO implant removal after surgery	8
Implant-associated infection	2
Polyarthritis	1
Premature physeal closure in the distal femur	1
Femoral osteochondritis dissecans	1
Femoral osteoproliferation	1
Pelvic limb amputation	1
Popliteal tendon avulsion	1
Failed tibial plateau leveling osteotomy	1
Femoral head ostectomy	1

**Note**: 55 dogs in total were excluded during case filtering because of confounding clinical factors. More than one confounding factor was found in some dogs.

The age of the study population was 5.3±2.7 years old and ranged from <1 to 11 years. Body weight was 40.5±15.1 kg and ranged from 12.4 to 83 kg. Included in the study were 2 males, 38 castrated males, 1 female, and 44 ovariohysterectomized females. A range of breeds was represented, with 67 pure breeds and 18 mixed breed dogs. The most common breeds were Labrador Retriever (n = 21), Golden Retriever (n = 11), Newfoundland (n = 6), and German Shepherd (n = 5). Other breeds included Bernese Mountain Dog, Chesapeake Bay Retriever, Great Dane, Rottweiler, Boxer, Mastiff, Shar Pei, Bulldog, Brittany Spaniel, Siberian Husky, Standard Poodle, and Wirehaired Pointing Griffon (listed from high to low frequency; each with <5 dogs, total n = 24). Labrador Retriever crosses were the most common type of mixed breed dog (n = 5). Among these 85 dogs, 45 dogs had left CR and 40 dogs had right CR at diagnosis. No dog had a history of traumatic injury.

### Radiography

**Figure 4:**
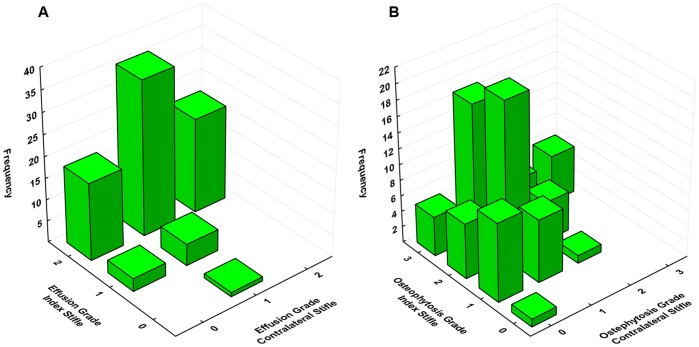
Relationship of osteoarthritic changes in the index and contralateral stifles. (**A**) Bivariate histogram of radiographic synovial effusion grade in index and contralateral stifles. (**B**) Bivariate histogram of osteophytosis grade in index and contralateral stifles. Severity of osteophytosis (S_R_ = 0.39, *p*<0.0005), but not synovial effusion (S_R_ = 0.17, *p* = 0.13) in the index and contralateral stifles were correlated.

In the index stifle, Grade 2 synovial effusion was found in 76 of 85 dogs (89%). Grade 2 effusion in the contralateral stifle was found in 22 of 85 dogs (26%). In the index stifle, Grade 3 osteophytosis was found in 34 of 85 dogs (40%). Grade 3 osteophytosis was found in 6 of 85 dogs (7%) in the contralateral stifle. In the index stifle, median severity of synovial effusion and osteophytosis was 2 (0,2) and 3 (0,3) respectively. In the contralateral stifle, median severity of synovial effusion and osteophytosis was 1 (0,2) and 1 (0,3) respectively. Severity of synovial effusion and osteophytosis was increased in the index stifle, when compared with the contralateral stifle (*p*<0.001). Severity of osteophytosis (S_R_ = 0.39, *p*<0.0005), but not synovial effusion (S_R_ = 0.17, *p* = 0.13) in the index and contralateral stifles were significantly correlated (**Fig. 4**). Body weight was not significantly correlated with either synovial effusion or osteophytosis in either the index or contralateral stifle. TPA in the index and contralateral stifles was 28±3 and 27±3 degrees respectively. There was no significance in TPA between index and contralateral stifles. TPA ranged from 21 to 35 in the index stifle and 19 to 34 in the contralateral stifle.

Intra-observer reproducibility for scoring of synovial effusion and osteophytosis on orthogonal stifle radiographs was 0.87 (95% confidence interval −0.73–0.95) and 0.82 (95% confidence interval −0.64–0.93) respectively. Inter-observer reproducibility for scoring of synovial effusion and osteophytosis was 0.83 (95% confidence interval −0.64–0.93) and 0.84 (95% confidence interval −0.67–0.94) respectively.

### Survival Analysis

Overall, 28 of 85 dogs (33%) developed contralateral CR within the study period. At the end of the study period of 2,516 days, 67% of dogs had not developed a contralateral CR. In the lower quartile of the cohort, time to contralateral CR was 498 days. Development of contralateral CR was significantly influenced by radiographic change in both stifles.

At one year (365 days) after diagnosis, development of contralateral CR was significantly influenced by severity of radiographic effusion in the contralateral stifle (*p* = 0.0001), but not the index stifle (**Tables 2** **–** **3**). A similar result was found at two years (730 days) after diagnosis (**Tables 2** **–** **3**). The odds ratio for development of contralateral CR in dogs with Grade 2 stifle effusion in the contralateral stifle was 13.4 by one year and 11.4 at two years after diagnosis. Similarly, at one year after diagnosis, development of contralateral CR was significantly influenced by severity of radiographic osteophytosis in the contralateral stifle (*p*<0.05), but not the index stifle (**Tables 4** **–** **5**). At two years after diagnosis, this effect was no longer significant (*p* = 0.11). The odds ratio for development of contralateral CR in dogs with Grade 3 osteophytosis in the contralateral stifle was 9.9 by one year after diagnosis.

**Table 2:** Relationship of radiographic synovial effusion in the index stifle to contralateral cranial cruciate ligament rupture at one and two years after surgery.

One year after surgery					
Effusion Grade	Ruptured	Not ruptured	Odds ratio	95% CI	Significance
0	0	1	1.25	0.05–31.98	*p* = 0.89
1	2	6	1.33	0.24–7.28	*p* = 0.74
2	15	59	0.89	0.17–4.73	*p* = 0.89
**Two years after surgery**					
0	0	1	0.61	0.02–15.72	*p* = 0.77
1	2	5	0.74	0.13–4.16	*p* = 0.73
2	20	36	1.67	0.31–9.04	*p* = 0.55

**Note**: At one year after surgery, n = 83 dogs; 2 dogs were censored at <365 days. Overall, radiographic synovial effusion in the index stifle was not significantly associated with risk of rupture at 1 year (*p* = 1.0). At two years after surgery, n = 64 dogs; 21 dogs were censored at <730 days. Overall, radiographic synovial effusion in the index stifle was not significantly associated with risk of rupture at 2 years (*p* = 1.0).

**Table 3:** Relationship of radiographic synovial effusion in the contralateral stifle to contralateral cranial cruciate ligament rupture at one and two years after surgery.

One year after surgery					
Effusion Grade	Ruptured	Not ruptured	Odds ratio	95% CI	Significance
0	1	19	0.15	0.02–1.25	*p* = 0.08
1	4	37	0.24	0.07–0.82	*p* = 0.02
2	12	10	13.44	3.88–46.51	*p* = 0.0001
**Two years after surgery**					
0	4	11	0.63	0.17–2.26	*p* = 0.47
1	6	27	0.21	0.07–0.65	*p* = 0.007
2	12	4	11.40	3.02–46.05	*p* = 0.0003

**Note**: At one year after surgery, n = 83 dogs; 2 dogs were censored at <365 days. Overall, radiographic synovial effusion in the contralateral stifle was significantly associated with risk of rupture at 1 year (*p* = 0.0001). At two years after surgery, n = 64 dogs; 21 dogs were censored at <730 days. Overall, radiographic synovial effusion in the contralateral stifle was significantly associated with risk of rupture at 2 years (*p* = 0.0001).

**Table 4:** Relationship of radiographic osteophytosis in the index stifle to contralateral cranial cruciate ligament rupture at one and two years after surgery.

One year after surgery					
Osteophytosis Grade	Ruptured	Not ruptured	Odds ratio	95% CI	Significance
0	0	1	1.25	0.05–31.98	*p* = 0.89
1	2	17	0.38	0.08–1.86	*p* = 0.23
2	6	25	0.89	0.29–2.72	*p* = 0.84
3	9	23	2.10	0.72–6.18	*p* = 0.18
**Two years after surgery**					
0	0	1	0.61	0.02–15.72	*p* = 0.77
1	3	14	0.32	0.08–1.25	*p* = 0.10
2	7	16	0.76	0.25–2.26	*p* = 0.62
3	12	11	3.38	1.14–10.00	*p* = 0.03

**Note**: At one year after surgery, n = 83 dogs; 2 dogs were censored at <365 days. Overall, radiographic osteophytosis in the index stifle was not significantly associated with risk of rupture at 1 year (*p* = 0.49). At two years after surgery, n = 64 dogs; 21 dogs were censored at <730 days. Overall, radiographic osteophytosis in the index stifle was not significantly associated with risk of rupture at 2 years (*p* = 0.09).

**Table 5:** Relationship of radiographic osteophytosis in the contralateral stifle to contralateral cranial cruciate ligament rupture at one and two years after surgery.

One year after surgery					
Osteophytosis Grade	Ruptured	Not ruptured	Odds ratio	95% CI	Significance
0	1	21	0.13	0.02–1.08	*p* = 0.06
1	10	34	1.34	0.46–3.96	*p* = 0.59
2	2	9	0.84	0.16–4.33	*p* = 0.84
3	4	2	9.85	1.63–59.51	*p* = 0.01
**Two years after surgery**					
0	3	14	0.32	0.08–1.25	*p* = 0.10
1	13	22	1.31	0.46–3.73	*p* = 0.61
2	2	5	0.74	1.13–4.16	*p* = 0.73
3	4	1	9.11	0.95–87.34	*p* = 0.06

**Note**: At one year after surgery, n = 83 dogs; 2 dogs were censored at <365 days. Overall, radiographic osteophytosis in the contralateral stifle was significantly associated with risk of rupture at 1 year (*p* = 0.03). At two years after surgery, n = 64 dogs; 21 dogs were censored at <730 days. Overall, radiographic osteophytosis in the contralateral stifle was not significantly associated with risk of rupture at 2 years (*p* = 0.11).

When data were analyzed using a univariate logistic regression model, development of contralateral CR at one year after diagnosis was significantly influenced by severity of radiographic effusion (odds ratio [unit change] = 4.7, *p*<0.005) and osteophytosis (odds ratio [unit change] = 2.1, *p* = 0.01) in the contralateral stifle. The effects of breed, age, body weight, gender, TPA, index effusion, and index osteophytosis (*p* = 0.08) were not significant. In the final multivariate model, development of contralateral CR was significantly influenced by radiographic effusion in the contralateral stifle (odds ratio [unit change] = 5.0), radiographic osteophytosis in the index stifle (odds ratio [unit change] = 2.0), and radiographic osteophytosis in the contralateral stifle (odds ratio [unit change] = 0.9) (*p*<0.001). Similar results were obtained when development of contralateral CR at two years after diagnosis was analyzed. In the two-year univariate model, risk of contralateral CR after diagnosis was significantly influenced by severity of radiographic osteophytosis in the index stifle (odds ratio [unit change] = 6.28, *p*<0.005) and severity of radiographic effusion in the contralateral stifle (odds ratio [unit change] = 2.07, *p*<0.05). The effects of breed, age, body weight, gender, TPA, index effusion, and contralateral osteophytosis (*p* = 0.06) were not significant. In the final multivariate model, development of contralateral CR was significantly influenced by radiographic effusion in the contralateral stifle (odds ratio [unit change] –2.60), radiographic osteophytosis in the index stifle (odds ratio [unit change] –2.78), and radiographic osteophytosis in the contralateral stifle (odds ratio [unit change] –0.74) (*p*<0.005).

Data were also analyzed using the Cox’s Proportional Hazards model. In the univariate model, development of contralateral CR after diagnosis was significantly influenced by severity of radiographic effusion (hazard ratio [unit change] = 2.4, *p*<0.005) in the contralateral stifle and osteophytosis (hazard ratio [unit change] = 1.64, *p* = 0.05) in the index stifle. The effects of breed, age, body weight, gender, TPA, index effusion, and contralateral OA (*p* = 0.08) were not significant. In the final multivariate model, development of contralateral CR was significantly influenced by radiographic effusion in the contralateral stifle (hazard ratio [unit change] = 2.8, *p*<0.005), radiographic osteophytosis in the index stifle (hazard ratio [unit change] = 2.0), but not radiographic osteophytosis in the contralateral stifle (hazard ratio [unit change] = 0.78, *p* = 0.37).

**Figure 5:**
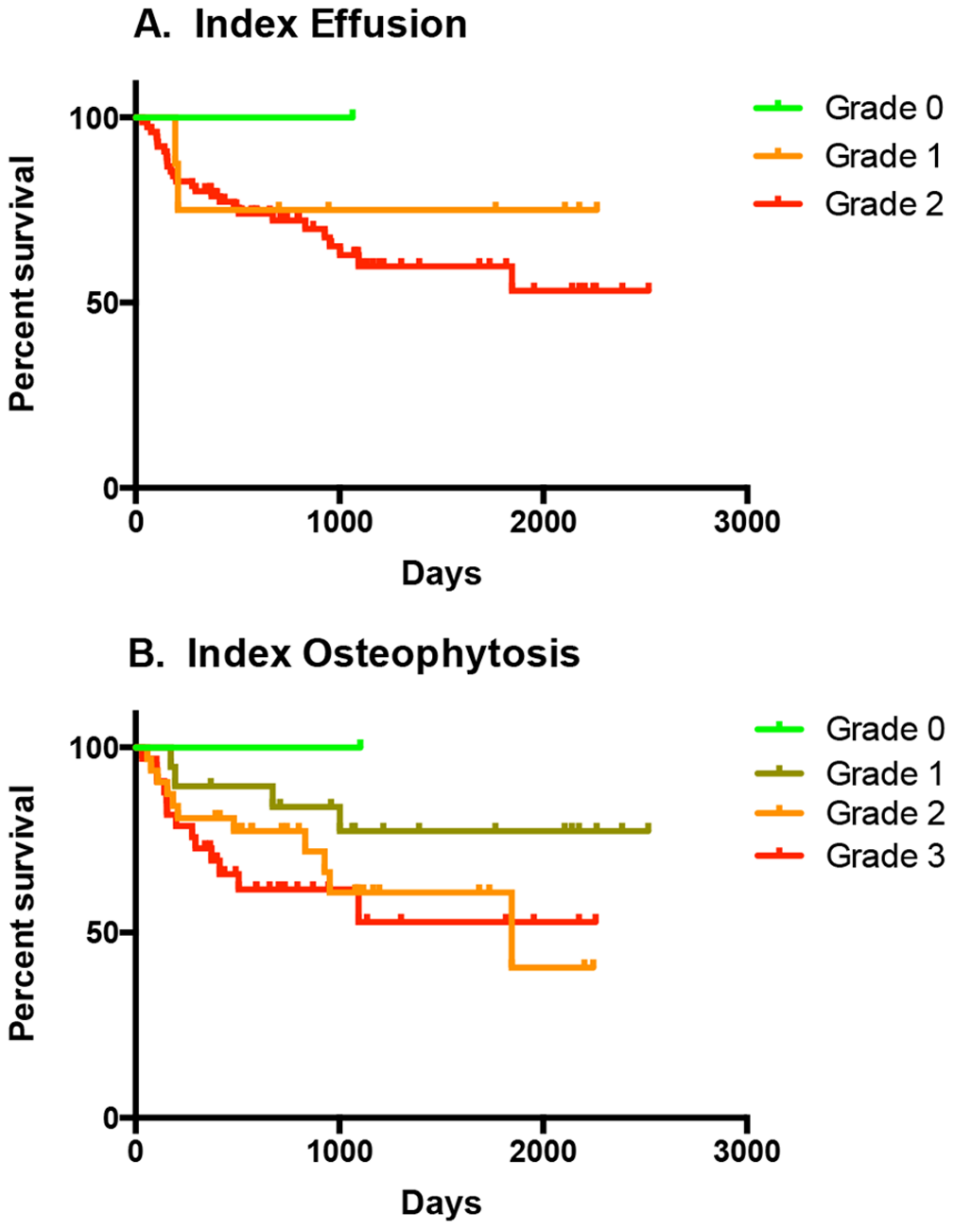
Time to contralateral cranial cruciate ligament rupture stratified by severity of synovial effusion and osteoarthritis in the index stifle. Kaplan-Meier plots for a population of 85 client-owned dogs. Time to contralateral cranial cruciate ligament rupture was not significantly influenced by severity of synovial effusion (**A**) or osteophytosis (**B**) in the index stifle.

**Figure 6:**
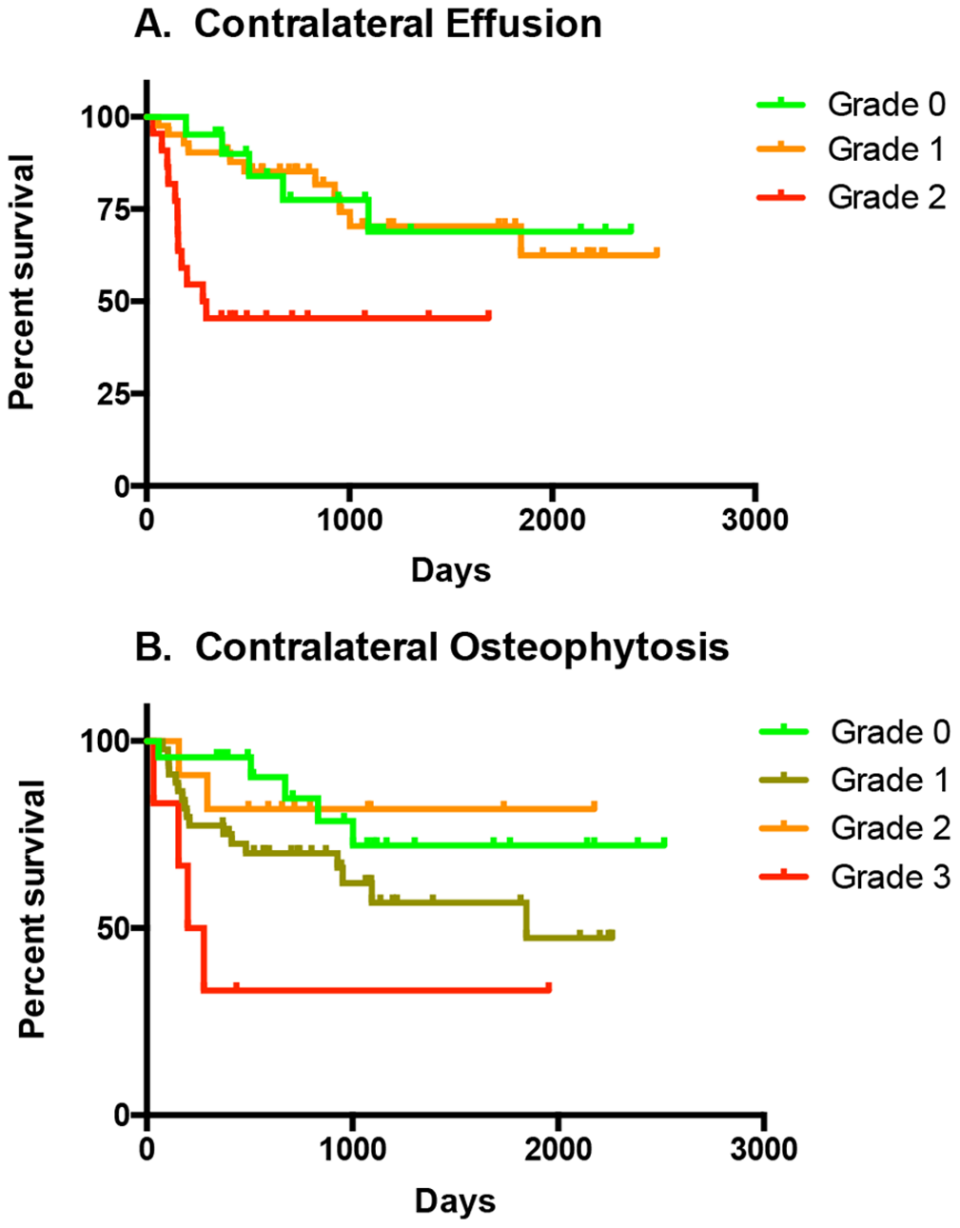
Time to contralateral cranial cruciate ligament rupture stratified by severity of synovial effusion or osteoarthritis in the contralateral stifle. Kaplan-Meier plots for a population of 85 client-owned dogs. (**A**) Time to contralateral cranial cruciate ligament rupture was significantly decreased in dogs with Grade 2 radiographic synovial effusion of the contralateral stifle at diagnosis, when compared with the grades of 0 or 1 (*p*<0.001). (**B**) Time to contralateral cranial cruciate ligament rupture was significantly decreased in dogs with Grade 3 osteophytosis of the contralateral stifle at diagnosis, when compared with the grade 0 and grade 2 (*p*<0.05).

When survival curves stratified by radiographic grading of severity of synovial effusion and osteophytosis were compared using the Logrank (Mantel-Cox) test, it was found that time to contralateral CR was not significantly influenced by severity of radiographic effusion or osteophytosis in the index stifle (**Fig. 5**). However, time to contralateral CR was significantly decreased in dogs with Grade 2 radiographic effusion of the contralateral stifle at diagnosis, when compared with the grades of 0 or 1 (*p*<0.001) (**Fig. 6A**). Time to contralateral CR was also significantly decreased in dogs with Grade 3 osteophytosis of the contralateral stifle at diagnosis when compared with the grades of 0 or 2 (*p*<0.05) (**Fig. 6B**).

## Discussion

Collectively, past observations suggest that the CR condition is a progressive, acquired degenerative condition in the dog. However, the disease mechanism remains poorly understood. Stifle synovitis develops in the initial phase of the condition and is often associated with some degree of fiber rupture in the cruciate ligament complex and an increased risk of subsequent contralateral CR [2], [12], [13]. Since radiography is routinely used for stifle joint evaluation in veterinary practice, the present study aimed to evaluate radiographic features for association with development of contralateral CR. We found that both effusion and osteophytosis of both stifles influenced the pattern of contralateral CR. Effusion of the contralateral stifle, in particular, was a highly significant risk factor for disease progression and development of contralateral CR, with an odds ratio of 13.4 for development of a contralateral CR by 1 year after diagnosis. We also found that radiographic scoring had excellent reproducibility, suggesting that it could successfully be used clinically.

It has been recognized for at least 25 years that dogs affected with unilateral CR often go on to develop contralateral CR. In a recent study, 11% of dogs were diagnosed with bilateral CR at diagnosis [11]. Risk of contralateral CR ranges from 22–54% at 6 to 17 months of diagnosis [6], [8]–[11]. The signalment of the cohort in the present study was typical for the condition and 33% of dogs developed contralateral rupture within the study period. Since this proportion was less than 50%, we were not able to define median time to contralateral CR. However, survival time for the lower quartile of the cohort was 498 days.

Past epidemiological studies have suggested that increasing age [23], neutering of male and female dogs [24], and breed [23] influence risk of CR. In addition, breeds at high risk of CR include the Newfoundland, Rottweiler, Labrador Retriever, Boxer, and Bulldog [23]. The CR trait has been shown to have moderate to high heritability in the Newfoundland and Boxer [25], [26]. In addition, proximal tibial conformation, including TPA, has also been considered an important risk factor for CR [27], [28]. In the present study, effects of breed, age, body weight, gender, and TPA did not significantly influence time to contralateral CR, in contrast to radiographic OA in the index and contralateral stifles, particularly the contralateral stifle. There is no evidence of homotypic variation in TPA in dogs [29] and very high TPA values were not present in our cohort in either the index or contralateral stifles. Whilst various epidemiological factors may be important for disease initiation, our results suggest that breed, age, body weight, gender, and TPA are not important factors affecting disease progression over time. These findings are similar to other recently published work [12]. Indirectly, this may suggest that risk factors for disease initiation and disease progression are different. The present study is not able to advance understanding of risk factors for disease initiation. Therefore, the relationship between initiation of fiber fraying in the cruciate ligament complex and development of contralateral stifle synovial effusion and osteoarthritis remains to be determined.

OA is usually detectable radiographically in CR-affected stifles at time of diagnosis [2], [14], [15]. We found Grade 2 effusion was present in a large majority of index stifles, with 40% of index stifles also affected with Grade 3 osteophytosis. Radiographic signs of contralateral stifle OA in dogs with unilateral CR influence risk of contralateral CR, based on global assessment of synovial effusion, osteophytosis, and subchondral sclerosis [6], [9]. However, past analyses have been limited to determining that global scoring of radiographic change, including synovial effusion and osteophytosis, influenced risk of contralateral CR. At diagnosis, we found that Grade 2 effusion was present in the contralateral stifle in 26% of dogs, with Grade 3 osteophytosis being identified in 7% of dogs. Severity of OA was increased in the index stifle. We also found that severity of stifle osteophytosis in the index and contralateral stifles was significantly correlated, suggesting that the disease mechanism influences OA progression in both stifles. Severity of radiographic synovial effusion and osteophytosis was not significantly correlated with body weight, suggesting body weight does not confound interpretation of radiographic change.

In a previous study, we used survival analysis of time to contralateral CR in a large group of dogs presented with unilateral CR to examine the pattern of contralateral CR [7]. The median time to contralateral CR for the entire affected population was 947 days [7]. However, we did not consider radiographic change at the time of diagnosis in the statistical model. Furthermore, various types of surgical treatment were provided that could have influenced overall mobility after surgery [16]–[18]; in the present study all dogs were treated with TPLO. In the current study, we found that both severity of radiographic synovial effusion and osteophytosis in the contralateral stifle were significant factors for contralateral rupture associated with a high odds ratio for contralateral CR, particularly at one year after diagnosis. Although a similar trend was identified at 2 years after diagnosis, analysis at this time point had some limitations because not all censored cases had two or more years of follow-up. Kaplan-Meir survival curves for severe Grade 2 radiographic synovial effusion and Grade 3 osteophytosis in the contralateral stifle had significantly reduced time to contralateral CR, when compared with dogs with less severe radiographic grades. Differences between Grade 3 and Grade 1 osteophytosis curves were not significantly different, likely because of low statistical power.

Results were not significant for radiographic synovial effusion in the index stifle, because Grade 2 effusion is typically present once ligament rupture is sufficient to induce joint instability. Survival analysis using logistic regression and the Cox’s Proportional Hazard model yielded similar results, although these analyses also suggested that severity of osteophytosis in the index stifle is a significant risk factor for contralateral CR.

It has been hypothesized that stifle synovitis may be an important factor contributing to development of the CR condition. It is known that synovitis is present in clinically stable stifles and that histologic synovitis increases the risk of subsequent contralateral CR [2], [13]. Results from the present study, particularly radiographic assessment of synovial effusion in the contralateral stifle, suggest that stifle synovitis is a significant factor in the mechanism that promotes progressive rupture of cruciate ligament fibers over time. This finding is not surprising, since experimental induction of stifle synovitis leads to a significant reduction in cranial cruciate ligament tensile properties [30], likely because the cruciate ligament complex is supplied by fluid flow from both the ligament vascular supply as well as stifle synovial fluid [31]. These findings suggest that the immune mechanisms that lead to stifle synovitis [4], [32] should be a focus for future work. Stifle synovitis likely represents a target for disease-modifying therapy aimed at blocking disease progression.

There were several limitations to this study. Our survival analysis was based on a group of 85 dogs treated with TPLO collected from a large cohort of client-owned dogs. However, many dogs were excluded in the initial case filtering, particularly because bilateral stifle radiographs at the time of diagnosis were lacking. This retrospective study reviewed case material over a nine-year period. During this period, it was not standard of care in small animal orthopaedic specialty practice to obtain bilateral stifle radiographs at the time of diagnosis. Consequently, a large number of cases were screened in order to yield an appropriate cohort of dogs for survival analysis. Although significant, results of some statistical analyses were associated with a relatively large confidence interval. Our conclusions may have been strengthened by analysis of longitudinal data from a larger number of dogs. Radiographic scoring was performed subjectively but yielded similar results to other work, in which synovial effusion or osteophytosis was graded as present or absent [12]. Our results strongly support the concept that bilateral radiographic views should be made routinely to fully assess both stifles for OA when dogs are suspected to have unilateral CR [12]. Furthermore, because of the retrospective nature of the study, there was some variation in the follow-up assessment that could have affected case coding. In particular, it is possible that owners may have failed to identify lameness associated with development of contralateral rupture.

In conclusion, subsequent contralateral CR is common in client-owned dogs. Radiographic synovial effusion and osteophytosis in the contralateral stifle of dogs with unilateral CR are significant, clinically relevant markers for risk of contralateral CR. Severe synovial effusion or osteophytosis in the contralateral stifle results in an approximately ten-fold increase in risk of contralateral CR. Our results suggest that stifle radiography provides a predictive marker for risk of contralateral CR that could be used to inform clinical treatment of affected dogs. Since stifle synovitis is a target for disease-modifying therapy, stifle radiography could also be a valuable tool for longitudinal studies of disease-modify treatment for the CR arthropathy. In future work, analysis of stifle magnetic resonance imaging would appear warranted, since synovitis, associated synovial thickening, and joint effusion can be more directly examined [33], [34].

## References

[1] Sumner JP, Markel MD, Muir P (2010) Caudal cruciate ligament damage in dogs with cranial cruciate ligament rupture. Vet Surg 39: 936–941. 20973805 10.1111/j.1532-950X.2010.00738.x

[2] Bleedorn JA, Greuel EN, Manley PA, Schaefer SL, Markel MD, et al (2011) Synovitis in dogs with stable stifle joints and incipient cranial cruciate ligament rupture: A cross-sectional study. Vet Surg 40: 531–543. 21615432 10.1111/j.1532-950X.2011.00841.x

[3] Wilke VL, Robinson DA, Evans RB, Rothschild MF, Conzemius MG (2005) Estimate of the annual economic impact of treatment of cranial cruciate ligament injury in dogs in the United States. J Am Vet Med Assoc 227: 1604–1607. 16313037 10.2460/javma.2005.227.1604

[4] Doom M, de Bruin T, de Rooster H, van Bree H, Cox E (2008) Immunopathological mechanisms in dogs with rupture of the cranial cruciate ligament. Vet Immunol Immunopathol 125: 143–161. 18621423 10.1016/j.vetimm.2008.05.023

[5] Comerford EJ, Smith K, Hayashi K (2011) Update on the aetiopathogenesis of canine cranial cruciate ligament disease. Vet Comp Orthop Traumatol 24, 91–98. 10.3415/VCOT-10-04-0055 21243176

[6] Doverspike M, Vasseur PB, Harb MF, Walls CM (1993) Contralateral cranial cruciate ligament rupture: incidence in 114 dogs. J Am Anim Hosp Assoc 29: 167–170.

[7] Muir P, Schwartz Z, Malek S, Kreines A, Cabrera SY, et al (2011) Contralateral cruciate survival in dogs with unilateral non-contact cranial cruciate ligament rupture. PLoS One 6: e25331. 21998650 10.1371/journal.pone.0025331 PMC3187768

[8] Moore KW, Read RA (1995) Cranial cruciate ligament rupture in the dog – a retrospective study comparing surgical techniques. Aust Vet J 72: 281–285. 8579557 10.1111/j.1751-0813.1995.tb03555.x

[9] de Bruin T, de Rooster H, Bosmans T, Duchateau L. van Bree H, et al (2007) Radiographic assessment of the progression of osteoarthrosis in the contralateral stifle joint of dogs with a ruptured cranial cruciate ligament. Vet Rec 161: 745–750. 18056011 10.1136/vr.161.22.745

[10] Cabrera SY, Owen TJ, Mueller MG, Kass PH (2008) Comparison of tibial plateau angles in dogs with unilateral versus bilateral cranial cruciate ligament rupture: 150 cases (2000–2006). J Am Vet Med Assoc 232: 889–892. 18341447 10.2460/javma.232.6.889

[11] Buote N, Fusco J, Radasch R (2009) Age, tibial plateau angle, sex, and weight as risk factors for contralateral rupture of the cranial cruciate ligament in Labradors. Vet Surg 38: 481–489. 19538670 10.1111/j.1532-950X.2009.00532.x

[12] Fuller MC, Hayashi K, Bruecker KA, Holsworth IG, Sutton JS, et al (2014) Evaluation of the radiographic infrapatellar fat pad sign of the contralateral stifle joint as a risk factor for subsequent contralateral cranial cruciate ligament rupture in dogs with unilateral rupture: 96 cases (2006–2007). J Am Vet Med Assoc 244: 328–338. 24432965 10.2460/javma.244.3.328

[13] Erne JB, Goring RL, Kennedy FA, Schoenborn WC (2009) Prevalence of lymphoplasmacytic synovitis in dogs with naturally occurring cranial cruciate ligament rupture. J Am Vet Med Assoc 235: 386–390. 19681718 10.2460/javma.235.4.386

[14] Bennett D, Tennant B, Lewis DG, Baughan J, May C, et al (1988) A reappraisal of anterior cruciate ligament disease in the dog. J Small Anim Pract 29: 275–297.

[15] Innes JF, Costello M, Barr FJ, Rudorf H, Barr ARS (2004) Radiographic progression of osteoarthritis of the canine stifle joint: a prospective study. Vet Radiol Ultrasound 45: 143–148. 15072147 10.1111/j.1740-8261.2004.04024.x

[16] Gordon-Evans WJ, Griffon DJ, Bubb C, Knap KM, Sullivan M, et al (2013) Comparison of lateral fabellar suture and tibial plateau leveling osteotomy techniques for treatment of dogs with cranial cruciate ligament disease. J Am Vet Med Assoc 243: 675–680. 23971847 10.2460/javma.243.5.675

[17] Nelson SA, Krotscheck U, Rawlinson J, Todhunter RJ, Zhang Z, et al (2013) Long-term functional outcome of tibial plateau leveling osteotomy versus extracapsular repair in a heterogenous population of dogs. Vet Surg 42: 38–50. 23153073 10.1111/j.1532-950X.2012.01052.x

[18] Bergh MS, Sullivan C, Ferrell CL, Troy J, Budsberg SC (2014) Systematic review of surgical treatments for cranial cruciate ligament disease in dogs. J Am Anim Hosp Assoc 50: epub. 10.5326/JAAHA-MS-6356 25028440

[19] Might KR, Bachelez A, Martinez SA, Gay JM (2013) Evaluation of the drawer test and the tibial compression test for differentiating between cranial and caudal stifle subluxation associated with cruciate ligament instability. Vet Surg 42: 392–397. 23231039 10.1111/j.1532-950X.2012.01064.x

[20] Cooper C, Cushnaghan J, Kirwan JR, Dieppe PA, Rogers J, et al (1992) Radiographic assessment of the knee joint in osteoarthritis. Ann Rheum Dis 51: 80–82. 1540044 10.1136/ard.51.1.80 PMC1004624

[21] Abel SB, Hammer DL, Shott S (2003) Use of the proximal portion of the tibia for measurement of the tibial plateau angle in dogs. Am J Vet Res 64: 1117–1123. 13677389 10.2460/ajvr.2003.64.1117

[22] Sham PC, Curtis D (1995) Monte Carlo tests for associations between disease and alleles at highly polymorphic loci. Ann Hum Genet 59: 97–105. 7762987 10.1111/j.1469-1809.1995.tb01608.x

[23] Witsberger TH, Villamil JA, Schultz LG, Hahn AW, Cook JL (2008) Prevalence of and risk factors for hip dysplasia and cranial cruciate ligament deficiency in dogs. J Am Vet Med Assoc 232: 1818–1824. 18598150 10.2460/javma.232.12.1818

[24] Whitehair JG, Vasseur PB, Willits NH (1993) Epidemiology of cranial cruciate ligament rupture in dogs. J Am Vet Med Assoc 203: 1016–1019. 8226247

[25] Wilke VL, Conzemius MG, Kinghorn BP, Macrossan PE, Cai W, et al (2006) Inheritance of rupture of the cranial cruciate ligament in Newfoundlands. J Am Vet Med Assoc 228: 61–64. 16426167 10.2460/javma.228.1.61

[26] Nielen ALJ, Janss LLG, Knol BW (2001) Heritability estimations for diseases, coat color, body weight, and height in a birth cohort of Boxers. Am J Vet Res 62: 1198–1206. 11497438 10.2460/ajvr.2001.62.1198

[27] Inauen R, Koch D, Bass M, Haessig M (2009) Tibial tuberosity conformation as a risk factor for cranial cruciate ligament rupture in the dog. Vet Comp Orthop Traumatol 22: 16–20. 19151865

[28] Mostafa AA, Griffon DJ, Thomas MW, Constable PD (2009) Morphometric characteristics of the pelvic limbs of Labrador retrievers with and without cranial cruciate ligament deficiency. Am J Vet Res 70: 498–507. 19335106 10.2460/ajvr.70.4.498

[29] Sabanci SS, Ocal MK (2014) Lateral and medial tibial plateau angles in normal dogs. Vet Comp Orthop Traumat 27: 135–140. 10.3415/VCOT-13-04-0043 24317701

[30] Goldberg VM, Burstein A, Dawson M (1982) The influence of an experimental immune synovitis on the failure mode and strength of the rabbit anterior cruciate ligament. J Bone Joint Surg 64A: 900–906. 7085718

[31] Kobayashi S, Baba H, Uchida K, Negoro K, Sato M, et al (2006) Microvascular system of the anterior cruciate ligament in dogs. J Orthop Res 24: 1509–1520. 16732615 10.1002/jor.20183

[32] Muir P, Kelly JL, Marvel SJ, Heinrich DA, Schaefer SL, et al.. (2011) Lymphocyte populations in joint tissues from dogs with inflammatory stifle arthritis and degenerative cranial cruciate ligament rupture. Vet Surg 40, 753–761. 10.1111/j.1532-950X.2011.00867.x 21770988

[33] Krasnokutsky S, Belitskaya-Lévy I, Bencardino J, Samuels J, Attuer M, et al (2011) Quantitative magnetic resonance imaging evidence of synovial proliferation is associated with radiographic severity of knee osteoarthritis. Arthritis Rheum 63: 2983–2991. 21647860 10.1002/art.30471 PMC3183134

[34] Hill CL, Gale DG, Chaisson CE, Skinner K, Kazis L, et al (2001) Knee effusions, popliteal cysts, and synovial thickening: association with knee pain in osteoarthritis. J Rheumatol 28: 1330–1337. 11409127

